# Lessons to Learn From 36 Cases of Well-Leg Compartment Syndrome in Colorectal Surgery: A Systematic Literature Review

**DOI:** 10.7759/cureus.67886

**Published:** 2024-08-27

**Authors:** Ali Yasen Mohamedahmed, Sangara Narayanasamy, Dakshita Agrawal, Marwa Yassin Mohamedahmed, Ashraf Fadul, Sadhasivam Ramasamy, Najam Husain, Pradeep Thomas

**Affiliations:** 1 General Surgery, Queen's Hospital Burton, University Hospitals of Derby and Burton NHS Trust, Burton-on-Trent, GBR; 2 Critical Care Medicine, Atbara Teaching Hospital, Atbara, SDN; 3 Orthopaedics and Traumatology, University Hospital Galway, Galway, IRL; 4 Colorectal Surgery, Queen's Hospital Burton, University Hospitals of Derby and Burton NHS Trust, Burton-on-Trent, GBR

**Keywords:** well-leg compartment syndrome, lloyd-davies position, fasciotomy, lithotomy position, colorectal surgery

## Abstract

Well-leg compartment syndrome is a rare and severe complication that occurs after prolonged surgery in the lithotomy position. This review outlines the presentation, diagnosis, and management of well-leg compartment syndrome after colorectal surgery. A comprehensive and systematic search of various electronic databases was conducted. All case reports and case series of well-leg compartment syndrome after colorectal surgery were included. Patient demographics, operative details, presenting symptoms, investigations, management, and treatment outcomes were collected from the eligible reports. Twenty-three articles, reporting a total of 36 patients, were eligible for inclusion in this review. Most of the included patients were male (88.9%), with an age range of 7-74 years. All reported cases in this review were placed in lithotomy position variations (standard lithotomy, Lloyd-Davies, and modified lithotomy) with an operative time exceeding four hours. Moreover, the presenting symptoms were lower limb pain, swelling, and loss of sensation on postoperative days 0 and 1. Fasciotomy was performed in 88.9% of cases, and half of the patients developed permanent sensory or motor dysfunction in the lower limbs. In conclusion, well-leg compartment syndrome is a rare, devastating complication that may result in permanent sensory or motor dysfunction. Early diagnosis and management are paramount for preserving limb function and optimising patient outcomes.

## Introduction and background

Well-leg compartment syndrome (WLCS) is a rare and severe complication that occurs after prolonged surgery in the lithotomy position. WLCS typically develops in legs that have not undergone any trauma, fracture, or injury, unlike acute compartment syndrome. This is a rare complication after surgery, with a reported incidence of 0.03-0.2%; however, it can significantly affect patients and must be recognised and treated immediately [1]. It most commonly occurs in gynaecological, colorectal, and urological surgeries where the lower limbs are placed in a lithotomy position [2]. The fascia, a vital structure within the lower limb that separates the muscles into different compartments, is a fixed connective tissue structure because its primary function is to support the leg [2]. The anterior compartment is most affected by compartment syndrome; however, there are also lateral and posterior compartments, further classified as deep posterior and superficial posterior compartments [1].

WLCS is associated with hypoperfusion that occurs in the legs owing to the lithotomy position of the legs during surgery. Prolonged elevation of the lower limb leads to decreased mean arterial flow to the legs, which can result in tissue ischemia [3]. Furthermore, ischemia leads to anaerobic respiration by producing acidosis, lactate, and other metabolites [1]. It can also negatively affect capillaries, damage blood vessels, and produce interstitial fluid, which increases inter-compartmental pressure. The increased compartment pressure further reduces the tissue arterial perfusion, indirectly increasing intra-compartmental pressure [4].

This review aims to highlight the clinical manifestations, diagnosis, and outcomes of reported cases of WLCS after colorectal surgery.

## Review

Methods

Two authors independently conducted a systematic search of online databases, including PubMed, MEDLINE, and Google Scholar, using relevant terms for WLCS case reports after colorectal surgery. The inclusion criteria were case reports or case series that reported WLCS after colorectal surgery. Age, sex, or language restrictions were not applied. Compartment syndrome cases after trauma or injury were excluded, as were cases reported after other types of surgery, such as gynaecological, orthopaedic, and vascular surgeries. Combinations of the following search terms were used: well-leg compartment syndrome, "well leg compartment syndrome", "non-traumatic compartment syndrome", "post-operative compartment syndrome", "colorectal surgery", "lithotomy position", colorectal surgery, and lithotomy position. Moreover, the reference lists of relevant studies were reviewed manually for potentially eligible studies.

Titles and abstracts of the selected articles were screened independently by two authors, and the full texts of potentially eligible articles were retrieved. Disagreements were resolved through consensus or consultation with the senior author. 

Two authors independently extracted data using an Excel spreadsheet. Information collected from each article included the author's name, year of publication, patient demographics, operation, patient position during the operation, operative time, clinical manifestation of WLCS, intervention, and outcome.

Results

**Figure 1:**
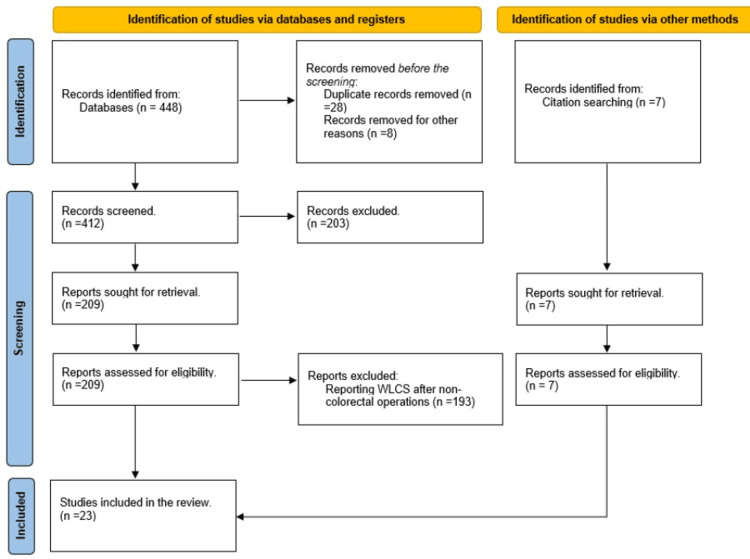
The PRISMA flowchart PRISMA: Preferred Reporting Items for Systematic Reviews and Meta-Analyses; WLCS: well-leg compartment syndrome

The systematic search yielded 450 articles, of which 234 were excluded after assessing the titles and abstracts because they were either duplicates or irrelevant to the review topic. Two hundred and sixteen studies were included in the full-text screening, of which 193 were excluded as they reported cases after operations other than colorectal surgery. Finally, 23 articles [5-27] reporting a total of 36 cases were deemed appropriate for inclusion in this review. Figure 1 shows the Preferred Reporting Items for Systematic Reviews and Meta-Analyses (PRISMA) flowchart.

Among the 36 reported cases, nearly 88.9% (32 patients) were male, and 11.9% (four patients) were female, with a wide age distribution between seven and 74 years (the mean was 44.3 years). Moreover, most reported cases were from Japan (13 cases) and the United Kingdom (11 cases). Indications for surgery in these reported cases were colorectal cancer (52.7%), inflammatory bowel disease (27.7%), and other indications (19.6%). The median operating time for these cases was 420 min (210-741 min).

Common presentations of WLCS in reported cases were lower limb pain, swelling, and loss of sensation on postoperative day (POD) 0 or POD 1. Delayed presentation of symptoms on POD 2 was reported in two cases [6,15], and in one case, symptoms were picked on POD 4 [22]. The criteria for diagnosing WLCS were clinical judgement, creatine kinase level (>2000 units/litre), lactate dehydrogenase (>280 units/litre), urine myoglobin (>10 milligrams per decilitre), and compartment pressure (>30 mmHg). Among the symptomatic patients diagnosed with WLCS, 88.9% underwent fasciotomy to relieve compartment pressure. Only a minority of 11.1% were treated conservatively. Regarding the outcome of treatment, 11 patients suffered a motor deficit, 10 patients had complete recovery, seven patients developed a sensory deficit, and six patients had chronic lower limb pain as a sequela of complications and treatment. Table 1 demonstrates the characteristics of the included cases. 

**Table 1:** Characteristics of reported cases of WLCS in the literature WLCS: well-leg compartment syndrome; NA: not available; M: male; BMI: body mass index; POD: postoperative day; min: minutes; CK: creatine kinase; U: unit; L: litre; ng: nanogram; UC: ulcerative colitis; FAP: familial adenomatous polyposis; MRI: magnetic resonance imaging

Author	Age and gender	BMI	Operation, position, and operative time (minutes)	Symptoms and diagnosis	Management and outcome
Arakawa and Sako 2023 [5]	51 M	25.9	Robotic anterior resection for rectal cancer, lithotomy, and head-down position for 360 min.	POD 0: Severe bilateral lower limb pain immediately after surgery. Diagnosis: Clinically.	Conservative management. Outcome: Complete recovery.
Crane et al. 2021 [6]	71 M	NA	Laparoscopic anterior resection for rectal cancer, Lloyd-Davies for 360 min.	POD 2: Left calf pain and swelling. Diagnosis: Clinically and high CK (3,780 U/L).	POD 2: Fasciotomy. Outcome: Weak dorsiflexion.
Lee et al. 2021 [7]	55 M	NA	Open abdominoperineal resection and cystectomy rectal cancer, Lloyd-Davies for 540 min.	POD 1: Pain in both lower limbs. POD 2: Diffuse and tense swelling. Diagnosis: Clinically.	POD 2: Fasciotomy. Outcome: Mobilisation with walking aid.
Sugi et al. 2021 [8]	56 M	25.5	Laparoscopic low anterior resection, Lloyd-Davies with Trendelenburg for 390 min.	POD 0: Pain and difficulty moving both legs. Diagnosis: Clinically, high CK (12,814 U/L) and leg compartment pressures.	POD 0: Fasciotomy. Outcome: Numbness in toes.
Nishino et al. 2018 [9]	46 M	28.1	Laparoscopic low anterior resection, lithotomy with Trendelenburg for 686 min.	POD 0: Calf pain and swelling. POD 1: Worsening pain and swelling. Diagnosis: Clinically, high CK (20,219 U/L) and urine myoglobin (26,000 ng/mL).	POD 1: Fasciotomy. Outcome: Mild muscle weakness.
Enomoto et al. 2016 [10]	28 M	28.9	Proctectomy and J pouch formation for UC, lithotomy position for 240 min.	POD 0: Pain, swelling, tenderness in both legs, and haematuria. Diagnosis: Clinically, high lactate dehydrogenase level (2103 U/L) and CK (142,850 U/L).	POD 0: Fasciotomy. Outcome: Mild sensory loss.
Konishi et al. 2016 [11]	52 M	34	Laparoscopic anterior resection for rectal cancer, Lloyd-Davies position for 328 min.	Diagnosed in POD 0.	POD 0: Fasciotomy. Outcome: Complete recovery.
Uetaki 2016 [9]	61 M	NA	Laparoscopic anterior resection for rectal cancer, Lloyd-Davies position for 741 min.	Diagnosed in POD 0.	POD 0: Fasciotomy. Outcome: Complete recovery.
Ozawa et al. 2015 [12]	61 M	25.9	Laparoscopic anterior resection for rectal cancer, Lloyd-Davies position for 385 min.	Diagnosed in POD 0.	POD 0: Fasciotomy. Outcome: Complete recovery.
Munekata et al. 2014 [13]	40 M	21.6	Laparoscopic anterior resection for rectal cancer, Lloyd-Davies position for 331 min.	Diagnosed in POD 1.	POD 1: Fasciotomy. Outcome: Complete recovery.
Uji 2014 [9]	68 M	NA	Laparoscopic anterior resection for rectal cancer, Lloyd-Davies position for 525 min.	Diagnosed in POD 0.	Conservative management. Outcome: Complete recovery.
Yoshimura and Mizumoto 2013 [14]	51 M	25.4	Laparoscopic anterior resection for rectal cancer, Lloyd-Davies position for 639 min.	POD 1: Leg pain and tenderness.	POD 1: Fasciotomy. Outcome: Sensory dysfunction.
Kalin et al. 2013 [15]	43 M	NA	Laparoscopic sigmoid colectomy and bladder repair, standard lithotomy position for 300 min.	POD 2: Loss of sensation, pain, and swelling of legs. Diagnosis: Clinically and high CK (8,000 U/L).	POD 2: Fasciotomy. Outcome: Transferred to plastic surgery.
Awab et al. 2012 [16]	55 F	27	Laparoscopic anterior resection for rectal cancer, Lloyd-Davies position for 720 min.	POD 0: Hot, painful left calf and dark-coloured urine. Diagnosis: Clinically and high CK (12,111 U/L).	Conservative treatment. Outcome: Complete recovery.
	74 M	24	Laparoscopic anterior resection for rectal cancer, Lloyd-Davies position for 600 min.	POD 0: Hot, painful left calf and dark-coloured urine. Diagnosis: Clinically and high CK (5,005 U/L).	Conservative treatment. Outcome: Complete recovery.
Takano et al. 2012 [17]	69 M	22.7	Laparoscopic anterior resection for rectal cancer, Lloyd-Davies position for 409 min.	POD 1: Leg pain and tenderness.	POD 1: Fasciotomy. Outcome: Sensory dysfunction.
Chin et al. 2009 [18]	44 F	26.6	Proctectomy, pouch formation, and ileostomy, Lloyd-Davies position for 420 min.	POD 0: Cramping-type pain in both legs. POD 2: Cold, mottled, and tender left calve. Diagnosis: Clinically and high CK (35,000 U/L).	POD 2: Fasciotomy. Outcome: Foot drop and mobilise with walking aid.
Krarup and Rawashdeh 2008 [19]	62 M	NA	Laparoscopic sigmoidectomy for sigmoid cancer, lithotomy position for 290 min.	Diagnosed in POD 1.	POD 1: Fasciotomy. Outcome: Sensory dysfunction.
Ikeya et al. 2006 [20]	67 M	29	Laparoscopic anterior resection for rectal cancer, lithotomy, and head-down position for 446 min.	POD 1: Bilateral lower limb pain and swelling. Diagnosis: Clinically, high CK (46,662 U/L) and MRI.	POD 1: Fasciotomy. Outcome: Complete recovery.
Wassenaar et al. 2006 [21]	34 M	24.2	Proctocolectomy and ileal pouch for FAP, modified lithotomy position for 420 min.	Pain in the lower leg. Diagnosis: Clinically and compartment pressure.	Fasciotomy. Outcome: Change job because of chronic leg pain.
	41 F	27.6	Ileoneorectal anastomosis for FAP, modified lithotomy position for 315 min.	Pain in the lower leg. Diagnosis: Clinically and compartment pressure.	Fasciotomy. Outcome: Change job because of chronic leg pain.
	39 M	31.4	Ileoneorectal anastomosis for UC, modified lithotomy position for 510 min.	Pain in the lower leg. Diagnosis: Clinically and compartment pressure.	Fasciotomy. Outcome: Change job because of chronic leg pain.
	23 M	22.1	Ileoneorectal anastomosis for UC, modified lithotomy position for 480 min.	Pain in the lower leg. Diagnosis: Clinically and compartment pressure.	Fasciotomy. Outcome: Chronic leg pain.
Dua et al. 2002 [22]	18 M	<30	Proctectomy and ileoanal pouch, lithotomy position for 210 min.	POD 4: Severe right leg pain, swelling, and loss of sensation. Diagnosis: Clinically.	POD 4: Fasciotomy. Outcome: Weak dorsiflexion.
	59 M	<25	Reversal of Hartmann's procedure, lithotomy position for 330 min.	POD 0: Tense left leg. Diagnosis: Clinically, compartment pressure.	POD 0: Fasciotomy. Outcome: Complete recovery.
Turnbull and Mills 2001 [23]	53 M	NA	Anterior resection for rectal cancer, Lloyd-Davies with Trendelenburg position for 240 min.	POD 0: Bilateral calf pain. Diagnosis: Clinically, high CK (>4,000 U/L).	POD 0: Fasciotomy. Outcome: Bilateral leg pain and numbness.
	54 M	NA	Ileoanal pouch formation for UC, Lloyd-Davies position for 420 min.	POD 0: Bilateral calf pain. Diagnosis: Clinically, high CK (>4,000 U/L) and compartment pressure.	POD 0: Fasciotomy. Outcome: Leg weakness and wheelchair bound.
Scott et al. 1997 [24]	26 M	NA	Completion of proctectomy and ileoanal pouch, Lloyd-Davies position for 540 min.	NA	Fasciotomy. Outcome: Complete recovery.
	18 M	NA	Completion of proctectomy and ileoanal pouch, Lloyd-Davies position for 480 min.	NA	Fasciotomy. Outcome: Right foot drop.
	35 F	NA	Pelvic dissection, resection of anastomotic recurrence, and coloanal anastomosis, Lloyd-Davies position for 660 min.	NA	Fasciotomy. Outcome: Webspace paraesthesia and fixed flexion deformity of the great toe.
	21 M	NA	Total colectomy and ileoanal pouch for UC, lithotomy position for 540 min.	NA	Outcome: Chronic calf pain.
Tuckey 1996 [25]	28 M	NA	Ileoanal pouch formation, Lloyd-Davies position for 455 min.	POD 1: Bilateral painful, tense, and swollen legs. Diagnosis: Clinically.	POD 1: Fasciotomy. Outcome: Bilateral foot drop.
Goldsmith and McCallum 1996 [26]	48 M	NA	Total colectomy and ileoanal pouch formation, Lloyd-Davies and Trendelenburg position for 420 min.	POD1: Hard, swollen, tender calves. Diagnosis: Clinically.	POD 1: Fasciotomy. Outcome: Bilateral foot drop.
	40 M	NA	Abdominoperineal resection rectal cancer, Lloyd-Davies position for 420 min.	POD 0: Pain, swelling, and erythema in the calves. Diagnosis: Clinically.	POD 0: Fasciotomy. Outcome: Complete recovery.
Peters et al. 1994 [27]	7 M	NA	Colon resection for residual Hirschsprung's disease, Lloyd-Davies position for 480 min.	POD 1: Painful swollen calf. POD 2: Weakness in both lower limbs. Diagnosis: Clinically.	POD 2: Fasciotomy. Outcome: Bilateral foot drop.
	59 M	NA	Reversal of Hartmann's procedure, Lloyd-Davies position for 390 min.	POD 0: Tender calves. Diagnosis: Clinically and high CK.	POD 1: Fasciotomy. Outcome: Bilateral foot drop.

Discussion

The main risk factors for WLCS are the position of the legs during surgery and the duration of surgery [2]. If the legs are placed in a lithotomy position, there is a greater risk of developing WLCS because the legs are above the level of the right atrium, which results in decreased perfusion of the lower limbs [28]. All reported cases in this review were placed in lithotomy position variations (standard lithotomy, Lloyd-Davies, and modified lithotomy) with an operative time exceeding four hours. There is a significantly increased risk of developing WLCS in surgeries that last more than four hours due to the patient remaining in a lithotomy position for a prolonged period of time [1,29]. Additionally, prolonged operative time reflects the complexity of the operation. Thus, these patients will have delayed recovery, which may mask the WLCS symptoms.

Furthermore, if the position of the head is also lowered, such as in the Trendelenburg position, leg perfusion is reduced, which additionally increases the risk of developing WLCS [30,31]. When the lower limbs are placed in the lithotomy position, the legs can be supported using Allen stirrups or knee supports. However, both of these supports have been shown to increase intra-compartmental pressure and are risk factors for developing WLCS [32]. The full dorsiflexed position of the ankle can also increase intra-compartmental pressure [33]. There are other factors, such as hypotension during surgery, intraoperative bleeding, peripheral vascular disease in patients [34], BMI >25 kg/m^2^ [2], and age <35 years, as they have stronger fascia [1].

Interestingly, compartment syndrome in the gluteal region following prolonged surgery has been described in the literature [35,36]. However, the presentation is similar to WLCS; it was described after surgical procedures in supine and prone positions. Gluteal compartment syndrome affects the three gluteal compartments and is associated with a higher rate of permanent neurological deficit due to the close proximity of the sciatic nerve [37].

Patients with WLCS commonly present within 24 hours after the effects of anaesthesia wear off. Patients typically complain of pain in the affected leg, difficulty in passive dorsiflexion of the toes and ankle, and tenderness on palpation; usually, the pain is not proportional to the surgery performed. Paraesthesia and swelling may also be present [3], and these symptoms have been identified in two case reports [38,9]. Most commonly, an ultrasound to explore deep vein thrombosis is performed, as it presents in a similar manner and is common after surgery. Often, the diagnosis is delayed as doctors are not familiar with this rare complication. The prompt diagnosis of WLCS is critical for optimising patient outcomes and minimising the risk of permanent disability. In this review, the majority of cases diagnosed and treated on POD 0 had either complete recovery or mild sensory dysfunction in comparison to those diagnosed on POD 1 or beyond.

WLCS is diagnosed by checking the delta pressure (diastolic blood pressure minus compartmental pressure), and if this is <30 mmHg, a diagnosis of WLCS should be suspected [1,38]. Another method of diagnosing WLCS is to check compartment pressure. If this pressure is greater than 30 mmHg, fasciotomy is required to release the leg compartments and save the affected limb. Normal compartment pressure during surgery should be 0-10 mmHg [34]. Moreover, creatinine kinase level >2000 units/litre is very unusual after surgery and indicates tissue damage due to diminished blood supply and necrosis [1,39].

Of the 36 included cases, 14 patients [6,8,9,11-14,16,17,19,20] had their operation laparoscopically, and one patient had a robotic-assisted procedure [5]. Recent advancements in minimally invasive surgery, such as laparoscopy and robotic-assisted surgery, have significantly improved surgical outcomes for colorectal cancer [40,41]. These approaches have been identified as potential risk factors for WLCS due to prolonged operative time [1,6,8]. Moreover, minimally invasive surgery requires certain patients to have access to the pelvic organs such as the rectum, bladder, and prostate. It has been reported that the incidence of WLCS in robotic radical prostatectomy is 0.29%, compared to only 0.03% in traditional open radical prostatectomy [42]. With the current shift toward minimally invasive surgery, WLCS incidence is expected to increase in the future.

WLCS is managed similarly to compartment syndrome caused by trauma and requires urgent referral to orthopaedic or vascular surgery [1]. Usually, a fasciotomy across the four compartments is performed to release compartmental pressure [2]. After 48-72 hours, the affected lower limb should be re-examined in the theatre for necrotic tissue and debridement of such tissue [1]. Four patients in this review were successfully treated using a non-operative approach [5,9,16]. Conservative treatment in these cases included leg elevation and intravenous rehydration along with clinical observation and serial creatine kinase. Conservative treatment has been described as ineffective for the management of traumatic compartment syndrome. However, it is a known treatment modality in chronic exertional compartment syndrome (CECS), a muscular overuse injury that usually affects athletes and military personnel [43]. 

Delayed recognition and management of WLCS increases the risk of tissue necrosis that can lead to amputation, Volkmann's contracture, and even death [3]. In addition, myoglobinuria may occur due to the release of myoglobin from muscle breakdown and rhabdomyolysis, which can adversely affect the kidneys, resulting in metabolic acidosis, renal failure, and, eventually, multiple organ failure [1-3]. Patients can also have residual symptoms post-compartment syndrome, and a study found that half of their study population had long-term neurological deficits and pain even two years after the diagnosis of compartment syndrome [44]. In the current review, three patients changed their jobs due to permanent disability resulting from WLCS [21]. Furthermore, in a case study, it was reported that pain post-WLCS was persistent after fasciotomy, resulting in the patient being diagnosed with chronic regional pain syndrome [38], which further emphasises the long-term complications of WLCS. 

**Figure 2:**
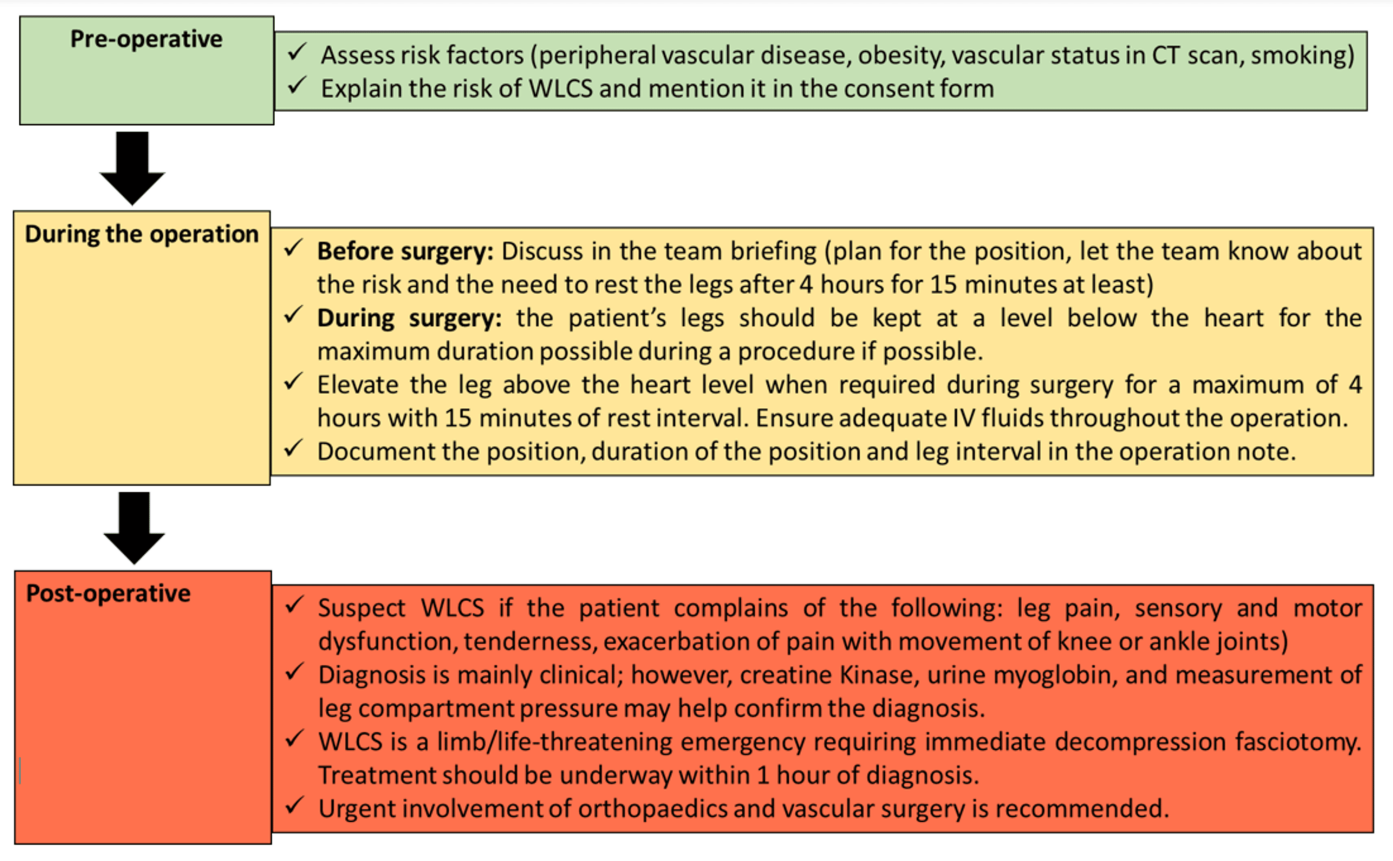
Algorithm demonstrating practical steps for the prevention, diagnosis, and management of WLCS. WLCS: well-leg compartment syndrome. Image Credits: Ali Yasen Mohamedahmed, Najam Husain, and Pradeep Thomas

Figure 2 provides an algorithm for the prevention and early recognition of WLCS. Prevention begins in the preoperative phase with a thorough assessment of risk factors and counselling for high-risk patients. Moreover, during a surgical briefing, it is essential to discuss the patient's position and the anticipated duration of the operation. Awareness among theatre teams is critical for mitigating the risk of WLCS. Early recognition involves continuous monitoring of the signs and symptoms of WLCS in the postoperative period. By implementing these perioperative strategies, surgeons can significantly reduce the incidence of WLCS, thereby enhancing patient safety and surgical outcomes.

Limitations 

This review is not without limitations. The included studies are either case reports or small case series, which carry an obvious risk of selection and reporting bias. This review exclusively includes case reports retrieved from online databases, which may not capture all instances of WLCS, particularly those reported in non-indexed journals or unpublished case series. Additionally, the reliance on published case reports may introduce reporting bias, as cases with negative outcomes or atypical presentations might be underreported. Moreover, several important details were not mentioned in the included reports, such as BMI, degree of fasciotomy, and whether the patient required repeated sessions of debridement. The lack of control groups in case reports and case series limits the ability to assess risk factors or evaluate the efficacy of specific treatment modalities. In summary, while this review offers valuable insights into WLCS following colorectal surgery, these limitations highlight the need for further research, preferably in the form of controlled studies or registries, to better understand the condition and optimise patient outcomes.

## Conclusions

WLCS is a devastating complication that may result in permanent sensory or motor dysfunction. Early diagnosis of WLCS is paramount for preserving limb function and optimising patient outcomes. Despite the challenges posed by its rarity and nonspecific presentation, clinicians must maintain a high index of suspicion and use appropriate diagnostic modalities to facilitate prompt recognition and intervention. Thus, healthcare providers can mitigate the risk of irreversible tissue damage, minimise the need for extensive surgical intervention, and improve the overall prognosis of patients with WLCS.
